# Xylitol exposure and cardiovascular risk

**DOI:** 10.1093/eurheartj/ehae730

**Published:** 2024-11-20

**Authors:** Bettina K Wölnerhanssen, Anne Christin Meyer-Gerspach, Arduino Arduini

**Affiliations:** Metabolic Research Group, St. Clara Research Ltd, Kleinriehenstrasse 43, CH-4058, Basel, Switzerland; Department of Clinical Research, Faculty of Medicine, University of Basel, Spitalstrasse 8/12, CH-4031, Basel, Switzerland; Metabolic Research Group, St. Clara Research Ltd, Kleinriehenstrasse 43, CH-4058, Basel, Switzerland; Department of Clinical Research, Faculty of Medicine, University of Basel, Spitalstrasse 8/12, CH-4031, Basel, Switzerland; R&D Department, CoreQuest Sagl, Lugano, Switzerland; Iperboreal Pharma Srl, Pescara, Italy

**This commentary refers to ‘Xylitol is prothrombotic and associated with cardiovascular risk’, by M. Witkowski *et al*., https://doi.org/10.1093/eurheartj/ehae244 and the discussion piece ‘Erythritol and xylitol and CVD risk: a growing concern’, by M. Witkowski and S.L. Hazen, https://doi.org/10.1093/eurheartj/ehae729; ‘Elevated serum xylitol levels and cardiovascular risk: an active component or an innocent bystander?’, by M. Bonomini *et al*., https://doi.org/10.1093/eurheartj/ehae731.**

Recently, Witkowski *et al.*^1^ provided observational data linking fasting plasma xylitol levels to the risk of cardiovascular (CV) events and *in vitro*, *in vivo* animal, and *ex vivo* human studies suggesting that xylitol may promote platelet activation and thrombus formation. The limitation of observational clinical studies is that, by design, they can only show association, not causation. Although xylitol is known as a sweetener, the findings in cohorts with CVD reflect endogenous synthesis, which is unrelated to consumption, as noted by the authors.^1^ Moreover, when reporting metabolomic data, usually no single metabolites, but clusters of metabolites are shown. Emerging evidence shows that various diseases are associated with an upregulation of endogenous polyol production. Unfortunately, the authors offer no hypothesis why xylitol production is increased in their cohorts, which might deepen our understanding of metabolic dysregulation.

The authors also report that *ex vivo* platelet response to stimulation with TRAP6 and ADP 30 min after ingestion of 30 g xylitol in 10 healthy humans was increased. However, with *n* = 10, the study was small; there was no placebo arm and only a single timepoint after ingestion. Short-term thrombocyte aggregation is e.g. altered in response to physical exertion and acute food intake. The single timepoint after 30 min does not allow drawing any conclusions about how relevant this observation is in the context of other natural fluctuations.

We therefore find the title of this publication misleading. It should say: ‘Endogenous xylitol production is elevated in patients with cardiovascular risk’ and ‘Xylitol intake might have a short-term effect on thrombocyte aggregation’.

Xylitol actually began its ‘career’ in Germany in the early 1970s, as a component of infusion products for critically ill patients. *BFARM* in Germany and *Pmda* in Japan approved a maximum i.v. dose of xylitol of 3 g/kg/day and 100 g/day, respectively, a stratospheric dose in comparison, which should have led to severe CV events. The only harmful but extremely rare side effect is the occurrence of severe secondary oxalosis after huge i.v. xylitol doses of >300 g/day, as recently reported in Japan in a patient who received a maximum dose of 500 g/day and a total dose of 2925 g in 8 days.^2^

In addition, alditols and monosaccharides (AME) from sorghum vinegar have been shown to inhibit several steps in the platelet aggregation pathway.^3^ Alditols and monosaccharides potently inhibited cyclooxygenase-1 (COX1) and thromboxane A2 synthase and attenuated thromboxane A2 production. Computational docking showed that alditols, such as erythritol and xylitol, could dock directly into the active site of COX. Bordier *et al.*^4^ have shown that 5 weeks of either erythritol or xylitol consumption had no significant effect on vascular function, abdominal fat, or glucose tolerance in obese people.

One final note, in the accompanying editorial,^5^ the authors misquoted: ‘… Very recent studies in humans show that transaldolase deficiency may cause dramatic accumulation of blood sugar alcohols (in the case of erythritol, several hundred-fold!) and may cause liver cancer.^10^’ Instead, the reference in number 10 cites a paper on the anticancer effects of xylitol, a kind of Freudian slip.

## Declarations

### Disclosure of Interest

B.K.W. and A.C.M.-G.: nothing to declare. A.A.: founder and major shareholder of CoreQuest and Iperboreal Pharma.

## References

[1] Witkowski M, Nemet I, Li XS, Wilcox J, Ferrell M, Alamri H, et al Xylitol is prothrombotic and associated with cardiovascular risk. Eur Heart J 2024;45:2439–52. 10.1093/eurheartj/ehae244 38842092 PMC11492277

[2] Takayasu S, Kamba A, Yoshida K, Terui K, Watanuki Y, Ishigame N, et al Secondary oxalosis induced by xylitol concurrent with lithium-induced nephrogenic diabetes insipidus: a case report. BMC Nephrol 2020;21:157. 10.1186/s12882-020-01814-9 32357847 PMC7195762

[3] Li J, Yu G, Fan J. Alditols and monosaccharides from sorghum vinegar can attenuate platelet aggregation by inhibiting cyclooxygenase-1 and thromboxane-A2 synthase. J Ethnopharmacol 2014;155:285–92. 10.1016/j.jep.2014.05.018 24877847

[4] Bordier V, Teysseire F, Drewe J, Madörin P, Bieri O, Schmidt-Trucksäss A, et al Effects of a 5-week intake of erythritol and xylitol on vascular function, abdominal fat and glucose tolerance in humans with obesity: a pilot trial. BMJ Nutr Prev Health 2023;6:264–72. 10.1136/bmjnph-2023-000764 PMC11009538 38618550

[5] Beer JH, Allemann M. Xylitol: bitter cardiovascular data for a successful sweetener. Eur Heart J 2024;45:2453–5. 10.1093/eurheartj/ehae252 38842099

